# Randomized clinical trial of the effect of intraoperative humidified carbon dioxide insufflation in open laparotomy for colorectal resection

**DOI:** 10.1002/bjs5.50227

**Published:** 2019-11-17

**Authors:** J. Y. Cheong, B. Chami, G. M. Fong, X. S. Wang, A. Keshava, C. J. Young, P. Witting

**Affiliations:** ^1^ Colorectal Unit Concord Repatriation General Hospital, Concord Clinical School Concord New South Wales Australia; ^2^ Department of Pathology Sydney Medical School, University of Sydney Sydney New South Wales Australia

## Abstract

**Background:**

Animal studies have shown that peritoneal injury can be minimized by insufflating the abdominal cavity with warm humidified carbon dioxide gas.

**Methods:**

A single‐blind RCT was performed at a tertiary colorectal unit. Inclusion criteria were patient aged 18 years and over undergoing open elective surgery. The intervention group received warmed (37°C), humidified (98 per cent relative humidity) carbon dioxide (WHCO_2_ group). Multiple markers of peritoneal inflammation and oxidative damage were used to compare groups, including cytokines and chemokines, apoptosis, the 3‐chlorotyrosine/native tyrosine ratio, and light microscopy on peritoneal biopsies at the start (T_0_) and end (T_end_) of the operation. Postoperative clinical outcomes were compared between the groups.

**Results:**

Of 40 patients enrolled, 20 in the WHCO_2_ group and 19 in the control group were available for analysis. A significant log(T_end_/T_0_) difference between control and WHCO_2_ groups was documented for interleukin (IL) 2 (5·3 *versus* 2·8 respectively; *P* = 0·028) and IL‐4 (3·5 *versus* 2·0; *P* = 0·041), whereas apoptosis assays documented no significant change in caspase activity, and similar apoptosis rates were documented along the peritoneal edge in both groups. The 3‐chlorotyrosine/tyrosine ratio had increased at T_end_ by 1·1‐fold in the WHCO_2_ group and by 3·1‐fold in the control group. Under light microscopy, peritoneum was visible in 11 of 19 samples from the control group and in 19 of 20 samples from the WHCO_2_ group (*P* = 0·006). The only difference in clinical outcomes between intervention and control groups was the number of days to passage of flatus (2·5 *versus* 5·0 days respectively; *P* = 0·008).

**Conclusion:**

The use of warmed, humidified carbon dioxide appears to reduce some markers related to peritoneal oxidative damage during laparotomy. No difference was observed in clinical outcomes, but the study was underpowered for analysis of surgical results. Registration number: NCT02975947 ( http://www.clinicaltrials.gov/).

## Graphical abstract

Open abdominal surgery leads to peritoneal desiccation and damage, resulting in inflammation. This peritoneal damage can be mitigated by the use of warmed, humidified carbon dioxide.

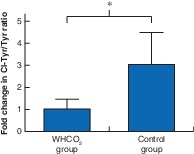

Peritoneal inflammation and open surgery

## Abstract (es)

**Antecedentes:**

Los estudios en animales han demostrado que la lesión peritoneal se puede minimizar insuflando gas de dióxido de carbono caliente y humidificado (*warm, humidified carbon dioxide gas*,WHCO_2(g)_) en la cavidad abdominal. El objetivo de este ensayo fue investigar los marcadores de inflamación peritoneal y de daño oxidativo en pacientes sometidos a cirugía colorrectal y abdominal tratados dióxido de carbono calentado humidificado en comparación con controles. El objetivo secundario fue evaluar los resultados clínicos perioperatorios.

**Métodos:**

Se llevó a cabo un ensayo aleatorizado, controlado y simple ciego en una unidad colorrectal terciaria. Se incluyeron pacientes de > 18 años de edad sometidos operaciones electivas por vía abierta. El grupo de intervención recibió CO_2(g)_ calentado (37°C) y humidificado (98% humedad relativa). Para la comparación de los grupos, se determinaron múltiples marcadores de inflamación peritoneal y daño oxidativo, incluyendo citocinas y quimiocinas, apoptosis (actividad Caspasas ‐3 y ‐7 y DeadEnd^TM^ TUNEl sistema fluorométrico), la tasa 3‐clorotirosina/tirosina nativa (HPLC‐MS) y microscopía electrónica de biopsias peritoneales al inicio (T_0_) y al término (T_final_) de la operación. Los resultados clínicos postoperatorios se compararon entre los grupos.

**Resultados:**

De los 40 pacientes incluidos en el estudio, se dispuso de datos para el análisis en 20 pacientes asignados al grupo de CO2 y en 19 asignados al grupo control. Se observó una diferencia significativa Log(Tend/T0) entre los grupos respecto a IL‐2 (grupo control: 5,34, grupo CO2: 2,78, *P* = 0,028) y IL‐4 (grupo control: 3,53, grupo CO2: 2,00, *P* = 0,04), en tanto que los análisis relativos a la apoptosis no pusieron de manifiesto cambios significativos en la actividad de la caspasa, y se observaron tasas de apoptosis similares a lo largo del borde peritoneal en ambos grupos. La tasa 3‐clorotirosina/tirosina nativa aumentó en 1,05 veces en el grupo del CO2 y en 3,1 veces en el grupo control. Por microscopía óptica el peritoneo era visible en el 57,9% de los sujetos del grupo control y en el 95% de los que recibieron tratamiento con WHCO_2(g)_ (*P* = 0,006). La única diferencia en los resultados clínicos entre los grupos de intervención y control fue el número de días para el paso de gases (2,5 en el grupo de CO2 *versus* 5,0 días en el grupo control, *P* = 0,008).

**Conclusión:**

El uso de WHCO_2(g)_ parece disminuir algunos de los marcadores relacionados con el daño peritoneal por estrés oxidativo durante la laparotomía. Aunque no se observaron diferencias en los resultados clínicos, el estudio no tenía la suficiente potencia para analizar los resultados quirúrgicos.

## Introduction

Open abdominal surgery is performed routinely for a number of diseases; however, it can be complicated by postoperative ileus, infection, anastomotic leak and, in the long term, bowel obstruction. Open surgery exposes the intestine to ambient air (20°C, 0–5 per cent relative humidity), which, combined with operating theatre negative air ventilation, has the potential to cause serosal/peritoneal desiccation^1^. Peritoneal desiccation leads to peritoneal inflammation and loss of barrier function, and increases the risk of infection^1^, ^2^, ^3^, ^4^. In addition, peritoneal inflammation can lead to adhesion formation and subsequent bowel obstruction^1^, ^2^. Bowel desiccation may also be a factor in delaying the return of bowel function after surgery. Moreover, exposure can lead to vasoconstriction of splanchnic blood flow to the intestine. Previous studies^2^, ^3^, ^4^ have indicated that desiccation and cooling of the peritoneum from open surgical wounds or the use of cold, non‐humidified carbon dioxide insufflation may stimulate oxidative stress in peritoneal mesothelial cells. Thus, desiccation of the peritoneum may lead to peritoneal inflammation, which may manifest as oxidative damage and reduced splanchnic blood flow, with associated long‐term consequences.

One pathway for mitigating bowel desiccation is the use of humidified, warmed carbon dioxide gas. Carbon dioxide is heavier (44 g/mol) and denser (1·97 kg/m^3^) than the other components of atmospheric air^5^ at standard temperature and pressure. Insufflated carbon dioxide therefore tends to sink to the base of the abdominal wound. Furthermore, carbon dioxide assists in maintaining heat by creating a localized greenhouse effect within the abdominal cavity, and is readily saturated to 100 per cent with sterile water, thereby acting to inhibit bowel desiccation^1^, ^2^, ^4^, ^6^, ^7^. A recently published study^8^ that examined the effect of warmed, humidified carbon dioxide in simulated open abdominal surgery in mice found that peritoneal tissue damage, as determined by cellular retraction, bulging and microvillus damage, was significantly reduced in animals receiving warmed, humidified carbon dioxide compared with that in the standard laparotomy group.

The research question of the present study was whether peritoneal damage and inflammation are elicited during open abdominal operation, and whether the use of warmed, humidified carbon dioxide inhibits peritoneal damage. The primary aim was to investigate several markers of peritoneal inflammation and oxidative damage at the beginning and end of the surgical procedures in patients treated with warmed, humidified carbon dioxide (WHCO_2_ group) and in controls. A secondary aim was to evaluate the perioperative clinical outcomes in both groups.

## Methods

A single‐blind RCT was performed at a tertiary colorectal unit in Sydney (Concord Repatriation General Hospital, University of Sydney). The study group received warmed (37°C), humidified (98 per cent relative humidity) carbon dioxide gas. The control group received current standard practice, and no gas was insufflated into the open laparotomy wound.

### Inclusion criteria

Patients recruited into the study were aged more than 18 years and scheduled for elective resection via a midline laparotomy. Surgical indications included: potentially curable colorectal carcinoma, polyposis syndrome, diverticular disease, rectal prolapse and inflammatory bowel disease. Patients were excluded if they had emergency surgery, laparoscopic surgery and/or presented with chronic obstructive pulmonary disease (COPD) requiring home oxygen, were carbon dioxide retainers, or if it was determined that their forced expiratory volume in 1 s (FEV1) was less than 1 litre, or predicted FEV1/forced vital capacity ratio was below 50 per cent.

### Primary and secondary outcomes

The primary outcome measure was an increased degree of peritoneal inflammation and damage from the beginning to the end of operation. This included changes in levels of inflammatory cytokines, measurement of peritoneal tissue apoptosis and oxidative damage, visualization of the injury to peritoneum via light microscopy and immunofluorescence. A secondary outcome measure was the perioperative clinical result.

### Patient cohort and randomization

Patients undergoing elective surgery at Concord Hospital were eligible for the study. Ethics board approval was obtained and the trial was registered in http://clinicaltrials.org (NCT02975947). All patients were screened before surgery, and the trial rationale and procedure were explained carefully. Patients were then given an ‘opt in’ participant information sheet, after which written informed consent was obtained before final enrolment. Patients were randomized to the intervention and control arm by random sequence, generated using an online tool (http://www.random.org)^9^. Patient allocation to specific groups was concealed in opaque numbered envelopes and kept in a central location to be opened at the time of surgery. Both patients and investigators performing the analyses were blinded to the allocation.

### The humidification system

HumiGard™ (Fisher & Paykel Healthcare, Auckland, New Zealand) was used to deliver warmed, humidified carbon dioxide with the gas diffuser positioned at the upper end of the laparotomy wound at a depth of approximately 4 cm from skin after the wound retractors had been placed. Insufflation of carbon dioxide was continued until the laparotomy wound had been closed. The carbon dioxide was delivered at a rate of 10 l/min, at a pressure of 4·5 bar from pressurized gas cylinders, and then passed through the HumiGard™ system.

### Clinical records

Details of patient demographics (age, sex, co‐morbidity, level of mobility, BMI and ASA grade) and intraoperative information including the procedure performed, duration of surgery, number of assistants, duration and volume of carbon dioxide administered, intraoperative blood transfusion, intraoperative complications, allocation to an enhanced recovery after surgery (ERAS) protocol, and stoma formation were collected and used for statistical analysis. Core body temperature was measured throughout the operation using a transoesophageal probe.

### Specimen collection and treatment

Two peritoneal biopsies (1 cm^2^) were taken at the beginning (T_0_) and end of the operation before wound closure (T_end_) from the hepatorenal angle. All specimens were stored in 1·5‐ml capped tubes (Eppendorf, Hamburg, Germany), snap‐frozen in liquid nitrogen and stored at −80°C until required for biochemical or histological assessment. One of the two T_0_ and T_end_ specimens was homogenized. Samples were thawed at 20°C and the tissue was transferred into a glass tube containing 1 ml of buffer A, which consisted of: phosphate‐buffered saline (Sigma‐Aldrich, Sydney, Australia), cOmplete™ EDTA‐free Protease Inhibitor Cocktail (Sigma‐Aldrich), EDTA (Sigma‐Aldrich) and the water‐miscible phenolic antioxidant butylated hydroxyl toluene (Acros Organics, Morris Plains, New Jersey, USA) to inhibit the artificial oxidative tissue processes. Preparations of buffer A were used for a maximum of 3 months, and subsequently replaced with freshly prepared solutions to ensure optimal antioxidant activity of constituents.

The tissue was homogenized using a matched rotating piston (set to operate at 500 r.p.m.) and matching Teflon (DuPont, Stevenage, UK)‐coated glass tube, as described previously for human^10^ and animal^11^ tissues. The specimen was ground by the rotating piston initially for 1 min, cooled on ice (4°C) and then homogenized for a further 1 min. Finally, the tissue homogenates were aliquoted into 1·5‐ml capped tubes and stored at −80°C until required for biochemical analysis.

### Markers of peritoneal inflammation and oxidative damage

#### *Tissue cytokines and chemokines*

Levels of inflammatory cytokines and chemokines were measured using a commercial enzyme‐linked immunosorbent assay kit (Human cytokine (16 plex)/chemokine (9 plex) – Stripwell Chemiluminescent kit; Quansys Biosciences, Logan, Utah, USA) according to the manufacturer recommendations^12^, ^13^; each peritoneal homogenate was assayed in duplicate. The analysis required a chemiluminescent imaging platform (ChemiDoc™ XRS; Life Science Research, Bio‐Rad Laboratories, Hercules, California, USA) to image the multiplex assay plates. Quantitative analysis was performed using Q‐View™ Software (Quansys Biosciences). Measurements were made of the mean fold increase in cytokine/chemokines from the samples taken at T_0_ and T_end_. The total protein concentration in each homogenate was determined using bicinchoninic acid protein analysis, and used to normalize all biochemical parameters in the corresponding homogenate. The mean T_0_ value in the control group was used as a reference point to calculate the fold increase in other groups (control group T_end_, WHCO_2_ group T_0_ and T_end_). In addition, to compare the change in level of cytokines between WHCO_2_ and control groups, the log of T_end_/T_0_ was used. T_end_ values were divided by T_0_ values, then log (base 2) was applied and the values for the two groups were compared.

#### *Apoptosis*

Cell apoptosis was measured by detection of active caspase‐3/7 bioluminescence, assayed in the stored tissue homogenates with Caspase‐Glo® 3/7 Assay (Promega, Madison, Wisconsin, USA)^14^. The log(T_end_/T_0_) values for WHCO_2_ and control groups were compared. A second marker of cell viability was determined using the DeadEnd™ Fluorometric TUNEL System (Promega). The extent of DNA fragmentation (a surrogate marker for apoptosis) was quantified by the measurement of green fluorescence intensity with fluorescence microscopy. A counterstain using DAPI (4′‐6‐diamidino‐2‐phenylindole) was performed and detected as blue fluorescence. Images were captured using Zeiss Axio AX10 light microscopy (Carl Zeiss, Oberkochen, Germany) with fluorescence camera AxioCam ICm 1 (Carl Zeiss). TUNEL (terminal deoxynucleotidyl transferase‐mediated dUTP nick‐end labelling)‐stained images were reviewed by five scientists/pathologists independently, who were all blinded to sample identity, and the results analysed.

#### *Estimation of peritoneal protein damage*

To assess peritoneal protein damage, the level of 3‐chlorotyrosine (Cl‐Tyr) and total native tyrosine (Tyr) in the peritoneal homogenates was measured by high‐pressure liquid chromatography with mass spectrometry (HPLC‐MS). Halogenated molecules can serve as specific markers for pathological oxidation as a limited number of reactive hypohalous acids participate in protein oxidation, including hypochlorous, hypobromous and hypothiocyanous acids^15^, ^16^, ^17^. The biological oxidant and antimicrobial agent hypochlorous acid is generated by myeloperoxidase^18^. Recent studies have demonstrated that the Cl‐Tyr/Tyr ratio is a specific marker for myeloperoxidase‐catalysed chlorination at sites of inflammation^15^. The relative ratio of Tyr and Cl‐Tyr were calculated for each patient sample at T_0_ and T_end_, and results for the control and WHCO_2_ treatment groups were compared. Proteins in the tissue homogenates were hydrolysed to individual free amino acids^19^. Hydrolysed analytes were then purified by solid‐phase extraction and analysed on an Agilent 1290 series UHPLC system tandem with 6460A triple quadrupole mass spectrometer (Agilent Technologies, Santa Clara, California, USA). Analytes within the mixture were separated using an Agilent Zorbax Eclipse XDB‐C18 (4·6 × 5 mm, 1·8 μm) column fitted with a UPLC Zorbax Eclipse XDB‐C18 (4·6 × 5 mm, 1·8 μm) guard column, and with a mobile phase A containing 0·1 per cent formic acid (v/v) and mobile phase B containing 0·1 per cent formic acid (v/v) in 90 per cent acetonitrile and 10 per cent water (v/v). The gradient was started at 2 per cent of mobile phase B and increased to 10 per cent at 3 min, continuing to increase to 95 per cent over 4 min and maintained for 1 min, and then returned to 2 per cent at 15 min for 1 min of equilibration. Tandem mass spectrometry was performed using electrospray ionization equipped with jet stream technology in the positive mode. The gas temperature was optimized at 350°C with a flow of 12 l/min, and the sheath gas was at 375°C with a flow of 11 l/min. Capillary voltage was 3500 MeV, and the nebulizer pressure was 25 p.s.i. All analytes were detected in multiple reaction monitoring mode with fragment voltage at 135 V, using nitrogen as the collision gas. For each analyte, one quantitative transition and one qualitative transition were monitored, including the internal standard. Data acquisition was performed using MassHunter B.07.01 (Agilent Technologies), and data analysis was conducted using the accompanied MassHunter Qualitative and Quantitative B.07.00 (Agilent Technologies).

#### *Light microscopy*

Peritoneal architecture was examined by light microscopy after staining with haematoxylin and eosin. This approach has been used previously to assess peritoneal effacement^20^, ^21^, ^22^, ^23^, ^24^. Images were obtained using a Zeiss Axio AX10 light microscope (Carl Zeiss) with a digital camera output (Zeiss Axiocam 105 color; Carl Zeiss). The stained images were assessed independently by five pathologists at the University of Sydney Medical School, all blinded to sample assignment; their results were combined, analysed and quantified.

### Postoperative outcomes

Postoperative outcomes, including postoperative pain (morphine equivalent daily dose score), duration of patient‐controlled analgesia use (measured in days), duration of hospital stay (measured in days), time to return to bowel function (flatus, stool), commencement of diet, clinical complications (persistent ileus (defined as: failure of the patient to eat, pass flatus or evacuate the bowel within 5 days of laparotomy), anastomotic leak, wound infection, unexpected return to theatre, unexpected readmission, and complications, graded according to Clavien–Dindo classification^25^) were collated and analysed. Patients were followed up for 60 days from the date of discharge, by telephone interviews as well as in day clinics.

### Statistical analysis

Sample size calculations were performed using the standard deviation of the measure of oxidative stress, Cl‐Tyr^26^, ^27^, ^28^, ^29^. For a power of 80 per cent, at 5 per cent two‐sided significance, the sample size was 40 patients.

Data were analysed using IBM SPSS® version 23 (IBM, Armonk, New York, USA) and GraphPad Prism® version 7.0 (GraphPad Software, La Jolla, California, USA). Continuous variables were tested using the D'Agostino–Pearson test. Groups were compared with *t* tests or two‐way ANOVA, using Tukey's multiple comparison test for parametric continuous variables and the Mann–Whitney *U* test for non‐parametric continuous variables (tissue cytokines and chemokines, and apoptosis assays). The level of significance for all tests was set at *P* < 0·050.

## Results

**Figure 1:**
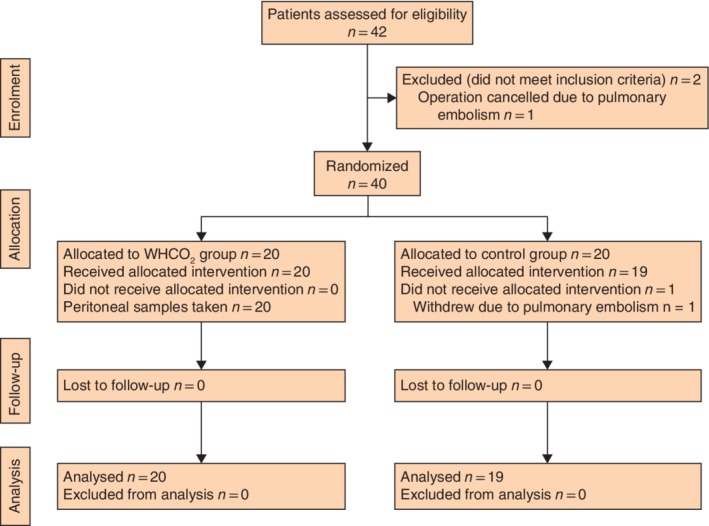
CONSORT diagram for the trial WHCO_2_, warmed, humidified carbon dioxide.

Between February 2013 and December 2016, 40 patients were recruited for the trial and randomly allocated into two groups, each of 20 subjects. However, one patient assigned to the control group withdrew from the study due to preoperative pulmonary embolism, leaving 20 patients in the WHCO_2_ group and 19 in the control group (*Fig*. 1). All data for the 39 patients were collected.

### Demographics

Of the 39 patients enrolled in the study, 15 were women and the mean age was 60·9 (95 per cent c.i. 55·9 to 66·0; range 32–87) years (*Table* 1). Mean BMI was 26·5 (95 per cent c.i. 24·1 to 28·9; range 14·2–43·1) kg/m^2^. Seventeen patients were overweight or obese (BMI above 25 kg/m^2^). Nine patients were smokers. There were no significant differences between the groups in age, sex, obesity, background medical history (including ischaemic heart disease, diabetes, hypertension, COPD and chronic renal failure), ASA fitness grade, smoking status, mobility, preoperative haemoglobin level and coagulation level (measured as the international normalized ratio). Eighteen patients had surgery for malignancy, six in the control group and 12 in the WHCO_2_ group, but there was no difference in the indication for surgery (*P* = 0·075).

**Table 1:** Demographics of patients in the carbon dioxide and control groups

	Control group (*n* = 19)	Carbon dioxide group (*n* = 20)	*P* *
**Age (years)**			
Mean	60·5	61·4	0·863†
Median (i.q.r.)	55·0 (48·0–77·5)	61·5 (56·5–70·7)	
**Sex ratio (M** : **F)**	(12 : 7)	(12 : 8)	0·839
**BMI (kg/m** ^**2**^ **)**			
Mean	27·7	25·4	0·334†
Median (i.q.r.)	27·6 (21·1–34·4)	23·0 (21·0–26·6)	
**BMI > 25 kg/m** ^**2**^	10	7	0·130
**IHD**	2	4	0·339
**COPD**	0	1	0·299
**Mean eGFR (ml per min per 1·73 m** ^**2**^ **)**	73·4	81·9	0·124†
**Diabetes mellitus**	4	5	0·640
**Hypertension**	7	10	0·267
**Any co‐morbidity**	8	12	0·148
**No. of co‐morbidities**			
Mean	0·7	1·1	0·332†
Median (i.q.r.)	0 (0–1·5)	1 (0–2)	
**ASA grade**			0·600
I–II	7	9	
III–IV	12	11	
**Preoperative haemoglobin level (g/dl)**			
Mean	130·7	125·1	0·485†
Median (i.q.r.)	137 (116·5–151·0)	128 (117·5–136·5)	
**Preoperative INR**			
Mean	1·0	1·1	0·562†
Median (i.q.r.)	1 (1·0–1·2)	1 (1·0–1·1)	
**Anticoagulants**	4	5	0·770
**Smoker**	6	3	0·219
**Mobility (4 flights of stairs)**	14	12	0·365
**Pathology/indication for surgery**			
Malignant disease	6	12	0·075
Benign disease	13	8	
ECF/parastomal hernia	3	1	
Reversal stoma	3	5	
IBD	4	2	
Diverticular disease	1	0	
Bowel fistula	2	0	

IHD, ischaemic heart disease; COPD, chronic obstructive pulmonary disease; eGFR, estimated glomerular filtration rate; INR, international normalized ratio; ECF, enterocutaneous fistula; IBD, inflammatory bowel disease.

* Pearson's χ^2^ test, except

† Student's *t* test.

### Surgical procedures

Procedures included abdominoperineal, high anterior, low anterior and ultralow anterior resections (*Table* 2). Other procedures included reversal of Hartmann's procedure, repair of enterocutaneous fistula, repair of parastomal hernia, pelvic exenteration, and resection of retroperitoneal colorectal cancer recurrence.

**Table 2:** Surgical procedures performed

	Control group	Carbon dioxide group
Rectal resection	8	4
Ileocolic resection	3	4
Small bowel resection	1	1
Total or subtotal colectomy	1	2
Other procedure	8	10

Some patients had more than one resection during the same operation.

### Inflammatory cytokines and chemokines

**Figure 2:**
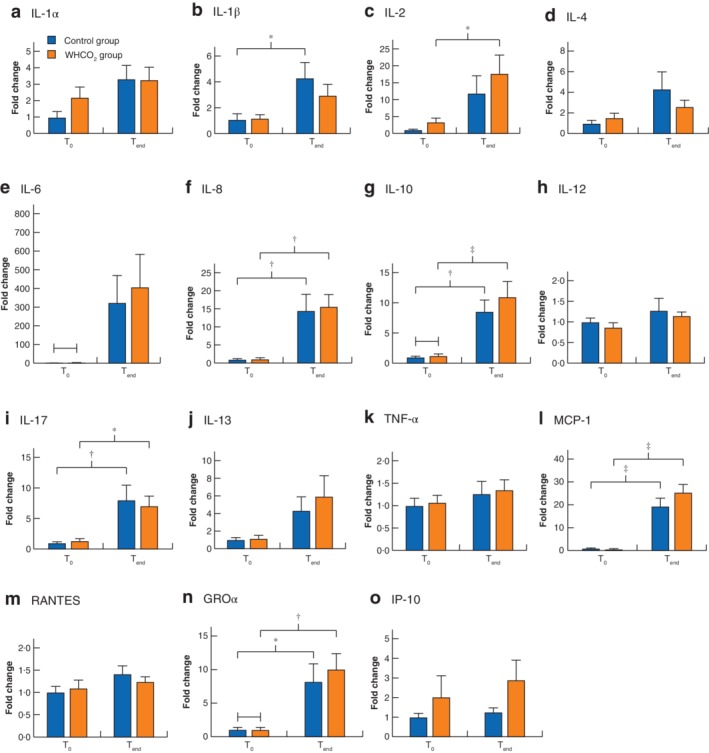
Change in levels of cytokines/chemokines in carbon dioxide and control groups Fold change in inflammatory cytokines and chemokines at the start (T_0_) and end (T_end_) of the operation in control and warmed, humidified carbon dioxide (WHCO_2_) groups: **a** interleukin (IL) 1α; **b** IL‐1β; **c** IL‐2; **d** IL‐4; **e** IL‐6; **f** IL‐8; **g** IL‐10; **h** IL‐12; **i** IL‐17; **j** IL‐13; **k** tumour necrosis factor (TNF) α; **l** monocyte chemotactic protein (MCP) 1; **m** RANTES (regulated on activation, normal T‐cell expressed and secreted); **n** growth‐regulated oncogene (GRO) α; **o** inducible protein (IP) 10. Values are mean(s.e.m.). *P < 0·050, †*P* < 0·010, ‡*P* < 0·001 (ANOVA using Tukey's multiple comparison test).

Levels of inflammatory cytokines and chemokines increased from the beginning to the end of the operation. When WHCO_2_ and control groups were combined and analysed together, a significant increase was observed from T_0_ to T_end_ for interleukin (IL) 1α, IL‐1β, IL‐2, IL‐4, IL‐5, IL‐6, IL‐8, IL‐10, IL‐13, IL‐15, IL‐17, monocyte chemotactic protein (MCP) 1, RANTES (regulated on activation, normal T‐cell expressed and secreted) and growth‐regulated oncogene (GRO) α (*Table* 3). There was no significant difference between the groups in any cytokine/chemokine level at T_0_ (*Fig*. 2). In the control group, a significant increase was seen from T_0_ to T_end_ in IL‐1β (4·2‐fold; *P* = 0·026), IL‐8 (14·5‐fold; *P* = 0·006), IL‐10 (8·5‐fold; *P* = 0·006), IL‐17 (8·0‐fold; *P* = 0·006), MCP‐1 (19·3‐fold; *P* < 0·001) and GROα (8·2‐fold; *P* = 0·037). In the WHCO_2_ group, there were significant increases for IL‐2 (from 3·5‐ to 17·8‐fold; *P* = 0·047), IL‐8 (from 1·2‐ to 15·5‐fold; *P* = 0·004), IL‐10 (from 1·3‐ to 11‐fold; *P* = 0·004), IL‐17 (from 1·4‐ to 7·1‐fold; *P* = 0·045), MCP‐1 (from 0·9‐ to 25·4‐fold; *P* < 0·001) and GROα (from 1·0‐ to 10·0‐fold; *P* = 0·003) (*Fig*. 2). In contrast, comparison of the fold change in inflammatory cytokines and chemokines for the control and WHCO_2_ groups using log(T_end_/T_0_) found statistically significant differences only for IL‐2 (5·3 *versus* 2·8; *P* = 0·028) and IL‐4 (3·5 *versus* 2·0; *P* = 0·041) (*Table* 4).

**Table 3:** Fold change increases in cytokine/chemokine levels

	Fold increase from T_0_ to T_end_	*P* *
IL‐1α	2·1	0·009
IL‐1β	3·2	0·002
IL‐2	6·0	< 0·001
IL‐4	2·4	0·021
IL‐5	3·0	0·002
IL‐6	135·2	< 0·001
IL‐8	13·8	< 0·001
IL‐10	8·3	< 0·001
IL‐12	1·3	0·120
IL‐13	4·4	0·004
IL‐15	4·2	0·011
IL‐17	6·4	< 0·001
TNF‐α	1·3	0·149
IFN‐γ	1·0	0·902
MCP‐1	24·2	< 0·001
RANTES	1·2	0·031
GROα	7·9	< 0·001
IP‐10	1·5	0·234

IL, interleukin; TNF, tumour necrosis factor; IFN, interferon; MCP, monocyte chemotactic protein; RANTES, regulated on activation, normal T‐cell expressed and secreted; GRO, growth‐regulated oncogene; IP, inducible protein.

* Unpaired *t* test.

**Table 4:** Comparison of increase in cytokine/chemokines measured using log(T_end_/T_0_)

	Log(T_end_/T_0_)		*P* *
	Control group	Carbon dioxide group	
IL‐1α	2·6	1·4	0·178
IL‐1β	3·3	1·0	0·178
IL‐2	5·3	2·8	0·028
IL‐4	3·5	2·0	0·041
IL‐5	2·7	3·4	0·990
IL‐6	8·6	7·4	0·363
IL‐8	5·0	4·1	0·805
IL‐10	3·4	3·8	0·635
IL‐12	0·9	1·4	0·691
IL‐13	2·7	3·5	0·539
IL‐15	3·7	4·3	0·675
IL‐17	5·0	3·2	0·244
TNF‐α	1·6	1·3	0·973
IFN‐γ	0·7	2·1	0·417
MCP‐1	4·0	4·8	0·112
RANTES	0·4	0·4	0·961
GROα	3·1	3·8	0·258
IP‐10	0·4	0·9	0·232

IL, interleukin; TNF, tumour necrosis factor; IFN, interferon; MCP, monocyte chemotactic protein; RANTES, regulated on activation, normal T‐cell expressed and secreted; GRO, growth‐regulated oncogene; IP, inducible protein.

* Mann–Whitney *U* test.

### Apoptosis assays

**Figure 3:**
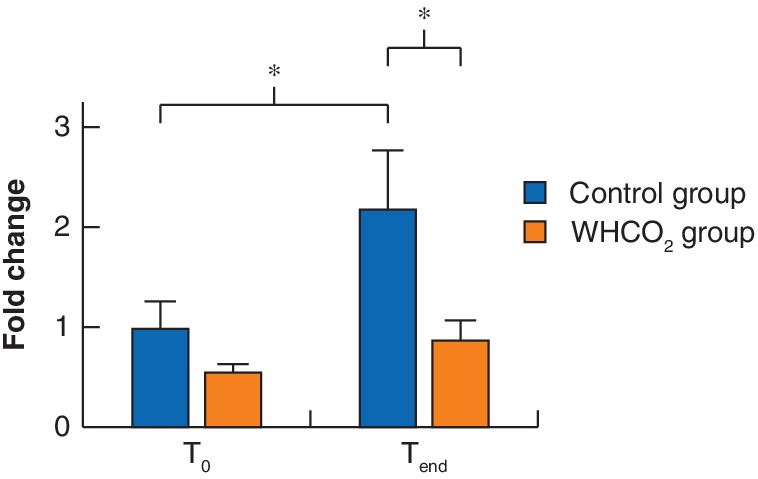
Change in degree of apoptosis in isolated peritoneal tissue Fold change in caspase‐3/7 activity in control and warmed, humidified carbon dioxide (WHCO_2_) groups at the start (T_0_) and end (T_end_) of the operation. Values are mean(s.e.m.). *P ≤ 0·050 (ANOVA using Tukey's multiple comparison test).

Apoptosis showed a twofold increase (95 per cent c.i. 1·1 to 2·9; *P* = 0·028) in the combined data cohort from T_0_ to T_end_. When considered separately, the control group showed a borderline increase in caspase‐3/7 activity (*P* = 0·050), whereas there was no significant change in caspase activity for the WHCO_2_ group (*P* = 0·883) (*Fig*. 3). However, analysis of the change in caspase‐3,7 activity by log(T_end_/T_0_) revealed no difference between control and WHCO_2_ groups (0·5 *versus* 1·2 respectively; *P* = 0·120).

**Figure 4:**
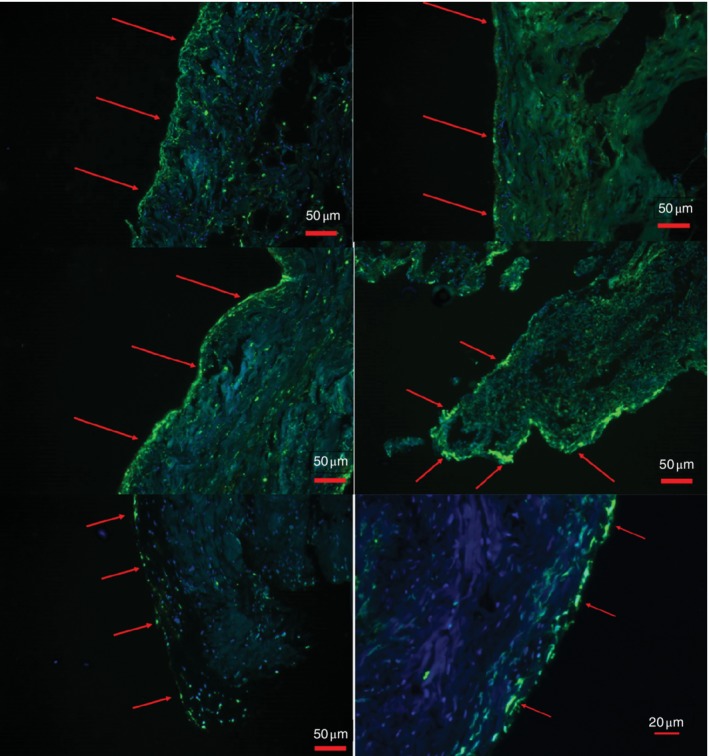
TUNEL assay of peritoneal edges Red arrows indicate areas of fluorescence green (apoptosis/necrosis) along the peritoneal edge. TUNEL, terminal deoxynucleotidyl transferase‐mediated dUTP nick‐end labelling.

*Fig*. 4 shows a diffuse green fluorescence and DAPI staining along the peritoneal edges of the section. In the combined cohort, cells comprising the peritoneal edges showed evidence of apoptosis in five of the 39 samples at T_0_, increasing to 23 of all 39 samples at T_end_. Comparison of the control and WHCO_2_ groups at T_end_ demonstrated that green fluorescence indicative of apoptosis was visible along the peritoneal edge in nine of the 19 control samples, a proportion slightly lower than the ten of 20 seen for the WHCO_2_ group (*P* = 0·869).

### Tissue 3‐chlorotyrosine/tyrosine ratio

The mean level of chlorinated tyrosine residues (as expressed by the Cl‐Tyr/Tyr ratio) was 4·3 for the WHCO_2_ group at T_0_, decreasing to 3·1 at T_end_. The corresponding evaluation for the control group revealed an increase in the ratio, from 2·3 at T_0_ to 3·6 at T_end_.

**Figure 5:**
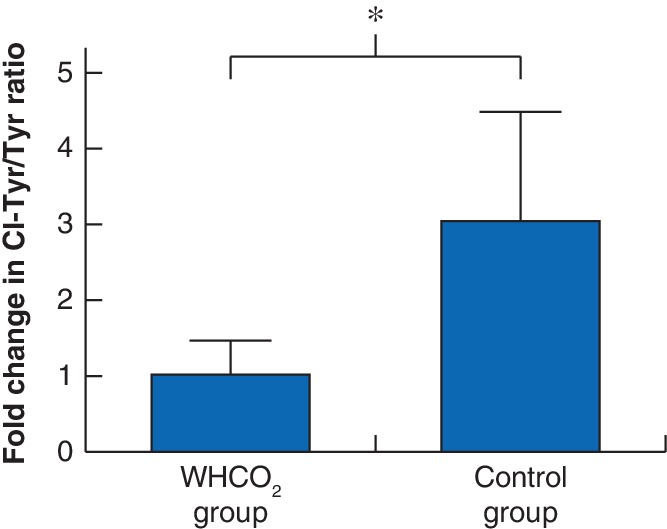
Change in level of oxidative damage in control and carbon dioxide groups Fold change in 3‐chlorotyrosine/tyrosine ratio (Cl‐Tyr/Tyr) from the start (T_0_) to the end (T_end_) of the operation in warmed, humidified carbon dioxide (WHCO_2_) and control groups. Values are mean(s.e.m.). *P < 0·050 (Mann–Whitney *U* test).

The relative levels of Cl‐Tyr/Tyr at T_end_ were re‐expressed as a fraction of those at T_0_: 3·1 (95 per cent c.i. 0·0 to 6·1) in the control group *versus* 1·1 (0·2 to 1·9) in the WHCO_2_ group (*P* = 0·036) (*Fig*. 5). Accordingly, for the WHCO_2_ group the Cl‐Tyr/Tyr ratio had increased by 1·1‐fold at T_end_, compared with the corresponding ratio measured at T_0_, whereas for the control group the ratio increased by a higher 3·1‐fold change from T_0_ to T_end_.

### Light microscopy

**Figure 6:**
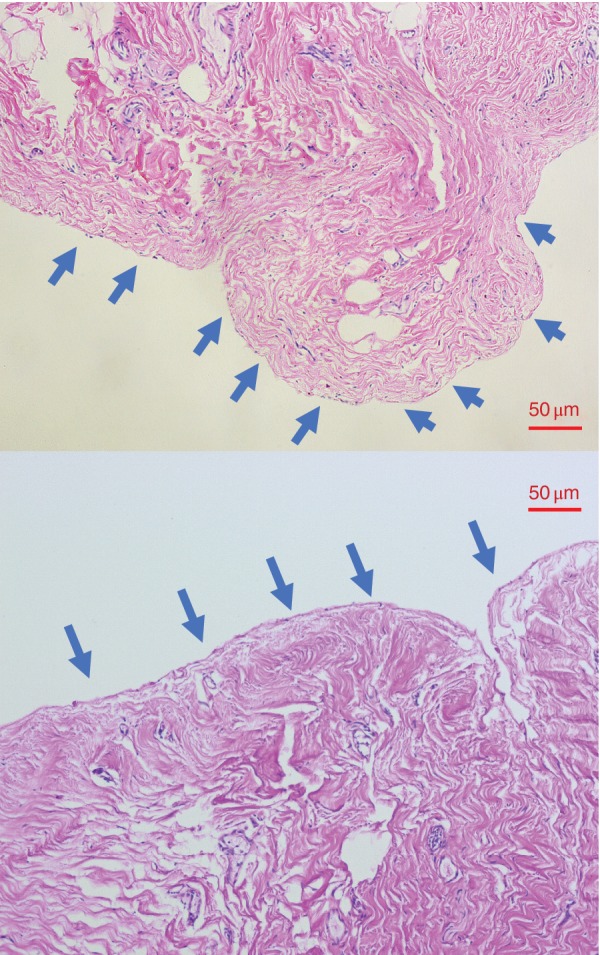
Visible peritoneum Haematoxylin and eosin‐stained section showing visible peritoneal edge (blue arrows).

**Figure 7:**
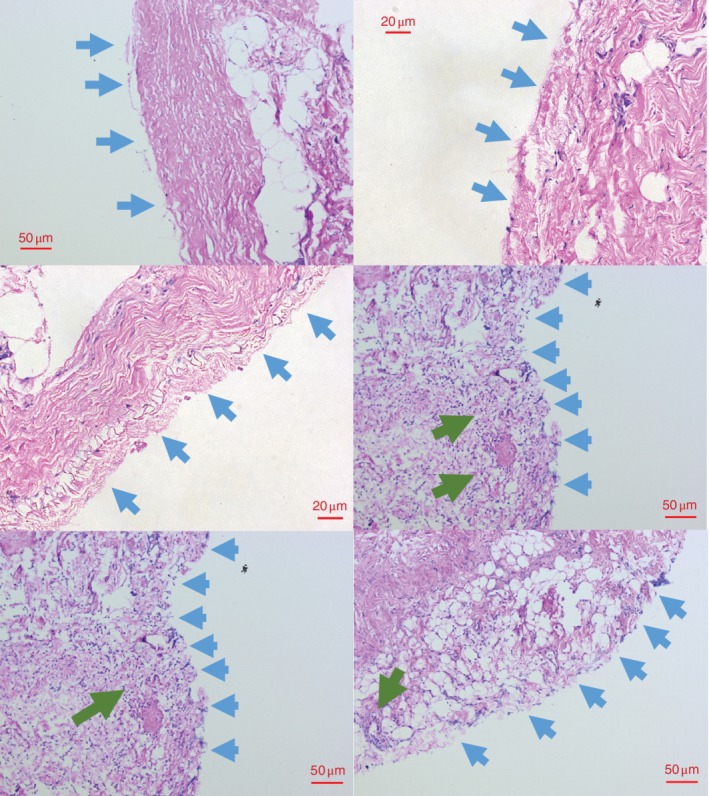
Peritoneal damage Haematoxylin and eosin‐stained sections showing denudation of the peritoneum (blue arrows). The last three images also show white cell infiltration of the peritoneum (green arrows).

Representative haematoxylin and eosin‐stained peritoneal images are shown in *Figs* 6 and 7. *Fig*. 6 shows visible peritoneum, whereas *Fig*. 7 shows denudation of the peritoneum with evidence of recruitment and infiltration of leucocytes. In the combined total cohort, peritoneum was visible in 36 of the 39 specimens collected at T_0_, whereas identification of visible peritoneum decreased to 30 of 39 at T_end_. By contrast, comparison of the control and WHCO_2_ groups at T_end_ revealed that peritoneum was visible in only 11 of 19 samples from the control group *versus* 19 of 20 samples in the WHCO_2_ group (*P* = 0·006).

### Surgical outcome

There were no significant differences between the control and WHCO_2_ groups with respect to duration of surgery, number of surgical assistants, intraoperative complications, stoma formation or number of patients assigned to the ERAS protocol (*Table* 5). Intraoperative blood transfusion was significantly greater in the WHCO_2_ group (6 of 20 patients *versus* 1 of 19 in the control group; *P* = 0·044).

**Table 5:** Summary of intraoperative and perioperative outcomes

	Total (*n* = 39)	Control group (*n* = 19)	Carbon dioxide group (*n* = 20)	*P* †
**Duration of surgery (min)** *	288·6	284·1	292·9	0·851‡
**No. of assistants** *	2·1	1·8	2·3	0·272‡
**Duration of exposure to carbon dioxide (min)** *		–	203·7	–
**Volume of carbon dioxide (litres)** *		–	2037	–
**Intraoperative blood transfusion**	7	1	6	0·044
**Intraoperative complications**	7	2	5	0·239
**ERAS protocol**	13	7	6	0·651
**Stoma formation**	14	4	10	0·060
End colostomy		1	3	
Loop ileostomy		2	4	
End ileostomy		1	3	
**Duration of hospital stay (days)** *	15·9	15·1	16·6	0·760‡
**Time to passage of flatus (days)** *	3·7	5·0	2·5	0·008‡
**Time to passage of stool (days)** *	4·6	5·5	3·7	0·092‡
**Commenced on clear/free fluids (days)** *	3·4	4·3	2·5	0·077‡
**Commenced on light diet (days)** *	5·9	6·9	4·9	0·125‡
**Unexpected return to theatre**	7	4	3	0·622
**Unexpected readmission**	7	3	4	0·732
**Postoperative ileus**	11	8	3	0·074
**30‐day mortality**	0	0	0	–
**Wound infection**	9	6	3	0·219
**Anastomotic leak**	1	0	1	–
**Clavien–Dindo complication grade**				
I	21	12	9	0·256
II	8	4	4	0·935
III–IV	9	4	5	0·770

* Values are mean. ERAS, enhanced recovery after surgery.

† Pearson's χ^2^ test, except

‡ Student's *t* test.

For the whole cohort, the mean duration of hospital admission was 15·9 (median 11, 95 per cent c.i. 10·6 to 21·2; range 3–77) days. Postoperative ileus occurred in 11 of the 39 patients. Wound infection developed in nine patients, and one of 30 patients with a bowel anastomosis subsequently developed an anastomotic leak. Seven patients had unexpected readmission after discharge from hospital: two for wound infection, one for a small bowel obstruction, one for perforated stomach, one for constipation due to narrowed colorectal anastomosis, one because of high‐output ileostomy dysfunction and one for a pelvic collection requiring drainage. No 30‐day mortality was reported (*Table* 5).

There was no difference between the two groups in duration of hospital stay or postoperative complications including wound infection, anastomotic leak, unexpected return to operating theatre and unexpected hospital readmission (*Table* 5). Patients in the WHCO_2_ group passed flatus significantly earlier than those in the control group (mean 2·5 *versus* 5·0 days respectively; *P* = 0·008).

## Discussion

Inflammation is a tightly controlled process with factors that are proinflammatory and anti‐inflammatory working simultaneously^30^. Whether localized peritoneal damage will promote a cascade of inflammation to the remaining bowel peritoneum is unclear; however, studies have shown that peritoneal damage is a feature occurring in animals and humans undergoing laparoscopy/laparotomy^30^.

Human and animal studies have found discrepant results regarding the effect of warmed, humidified carbon dioxide on peritoneal inflammation^30^. In laparoscopic human studies, it was reported^31^ that peritoneal damage was less with the use of warmed, humidified carbon dioxide gas insufflation, but another study^32^ documented no difference. Similarly, animal‐based studies have shown that use of warmed, humidified carbon dioxide resulted in less peritoneal damage and desquamation^21^, ^22^, ^33^, whereas other studies found no effect^23^, ^34^, ^35^. A recently published animal‐based study^8^ comparing the effect of warmed, humidified carbon dioxide during open abdominal surgery in mice *versus* passive airflow found significantly lower peritoneal tissue damage (as determined by cellular retraction, bulging, microvilli loss) in the WHCO_2_ group. In addition, peritoneal hypoxia, as measured by hypoxia‐inducible factor 1α concentration, was significantly lower in the WHCO_2_ group. This study^8^ also found that core body temperature was maintained better in the WHCO_2_ group.

The present study evaluated oxidative stress on the peritoneum during laparotomy by measuring Cl‐Tyr as a fraction of native tyrosine. Oxidative stress during laparoscopy has been described previously^36^, and has been suggested to be due in part to the pneumoperitoneum leading to an increase in intra‐abdominal pressure contributing to organ ischaemia with resultant formation of reactive oxygen species (ROS). These ROS then damage the membrane phospholipids, proteins and DNA, resulting in cellular injury. Other authors^36^ have hypothesized that, although raised intra‐abdominal pressure during laparoscopy (with resultant insufflation–deflation causing ischaemia–reperfusion injury) is contributory to oxidative stress, other factors could also contribute and interplay, such as anaesthesia, the surgical insult itself, patient position and the type of gas used. In addition, during laparotomy, where the peritoneum is exposed to the atmospheric conditions, factors contributing to oxidative stress could be desiccation, surgical insult and anaesthesia. Another systematic review^37^ found that the increase in oxidative stress was less in the laparoscopic than in the laparotomy group, despite both groups showing a marked increase in plasma levels of markers of oxidative stress. However, most of these studies were based on plasma‐based measurement of oxidative stress; only a few studies measured oxidative stress in tissue specimens (including tissue malondialdehyde concentration and gastric mucosal pH).

The reason for the greater oxidative stress observed in open surgery compared with laparoscopic procedures remains unclear. A possible explanation could be in relation to peritoneal dessication, with peritoneal dessication and activation of neutrophils leading to ROS damage to the peritoneum, which may be more marked in open surgery.

An important question derived from this study is whether the positive outcome of warmed, humidified carbon dioxide is sufficiently rigorous to demand translation into clinical practice. In this study the carbon dioxide treatment group achieved earlier passage of flatus, although the procedures were heterogeneous. Overall, the postoperative outcomes in the two groups were comparable and no other significant difference was noted. It would be interesting to note in future whether use of warmed, humidified carbon dioxide would result in a lower rate of adhesion formation. However, this study was neither designed nor powered to detect differences in adhesion formation between the intervention and control groups, nor to detect clinical difference.

Nonetheless, this trial in humans can act as a pilot for a larger study, as there were no significant technical drawbacks to the use of low‐level carbon dioxide infusion. Placement of the diffuser did not interfere with the operation and therefore had no impact on duration of surgery. Future studies involving warmed, humidified carbon dioxide could investigate its effect on intraoperative splanchnic blood flow. Warmed, humidified carbon dioxide would raise local temperature, prevent heat loss through convection, and lower local pH, thereby increasing bowel perfusion. This would be especially beneficial when bowel anastomosis was being performed, as adequate perfusion is essential for anastomotic healing. Examination of the inhibition of peritoneal oxidative damage by inhibition of myeloperoxidase may be another useful study.

## References

[1] Persson M , van Der Linden J. Intraoperative CO_2_ insufflation can decrease the risk of surgical site infection. Med Hypothesis 2008; 71: 8–13. 10.1016/j.mehy.2007.12.016 18304752

[2] Binder MM . Humidification during laparoscopic surgery: overview of the clinical benefits of using humidified gas during laparoscopic surgery. Arch Gynecol Obstet 2015; 292: 955–971. 25911545 10.1007/s00404-015-3717-y PMC4744605

[3] Tsuchiya M , Sato EF , Inoue M , Asada A . Open abdominal surgery increases intraoperative oxidative stress: can it be prevented? Anesth Analg 2008; 107: 1946–1952. 19020142 10.1213/ane.0b013e318187c96b

[4] van der Linden J , Persson M. CO_2_ field flooding may also reduce oxidative stress in open surgery. Anesth Analg 2009; 109: 683–684. 19608848 10.1213/ane.0b013e3181a90846

[5] http://reference.com. *Is CO* _*2*_ *Heavier Than Air?*; 2017 https://www.reference.com/science/co2-heavier-air-b69c3fe7a671fbad?qo=contentSimilarQuestions [accessed 12 October 2017].

[6] Persson M , Flock JI , Van der Linden J . Antiseptic wound ventilation with a gas diffuser: a new intraoperative method to prevent surgical wound infection? J Hosp Infect 2003; 54: 294–299. 12919760 10.1016/s0195-6701(03)00135-x

[7] Persson M , van der Linden J . Wound ventilation with carbon dioxide: a simple method to prevent direct airborne contamination during cardiac surgery? J Hosp Infect 2004; 56: 131–136. 15019225 10.1016/j.jhin.2003.10.013

[8] Carpinteri S , Sampurno S , Malaterre J , Millen R , Dean M , Kong J *et al* Experimental study of delivery of humidified–warm carbon dioxide during open abdominal surgery. Br J Surg 2018; 105: 597–605. 29193022 10.1002/bjs.10685 PMC5901019

[9] http://random.org. *Randomness and Integrity Services Ltd* https://www.random.org [accessed 3 September 2014].

[10] Raitakari OT , McCredie RJ , Witting P , Griffiths KA , Letters J , Sullivan D *et al* Coenzyme Q improves LDL resistance to *ex vivo* oxidation but does not enhance endothelial function in hypercholesterolemic young adults. Free Radic Biol Med 2000; 28: 1100–1105. 10832071 10.1016/s0891-5849(00)00201-x

[11] Witting P , Pettersson K , Ostlund‐Lindqvist AM , Westerlund C , Wâgberg M , Stocker R . Dissociation of atherogenesis from aortic accumulation of lipid hydro(pero)xides in Watanabe heritable hyperlipidemic rabbits. J Clin Investig 1999; 104: 213–220. 10411551 10.1172/JCI6391 PMC408476

[12] Trune DR , Larrain BE , Hausman FA , Kempton JB , MacArthur CJ . Simultaneous measurement of multiple mouse ear proteins with multiplex ELISA assay. Hear Res 2011; 275: 1–7. 21144888 10.1016/j.heares.2010.11.009 PMC3087854

[13] Quansys Biosciences . *Q‐Plex™ Manuals: Human Multiplex Assays. Human Cytokine – Stripwells* http://www.quansysbio.com/manuals [accessed 12 October 2017].

[14] Promega . *Technical Bulletin* *Caspase‐Glo® 3/7 Assay* https://www.promega.com/-/media/files/resources/protocols/technical-bulletins/101/caspase-glo-3-7-assay-protocol.pdf [accessed 12 October 2017].

[15] Talib J , Pattison DI , Harmer JA , Celermajer DS , Davies MJ . High plasma thiocyanate levels modulate protein damage induced by myeloperoxidase and perturb measurement of 3‐chlorotyrosine. Free Radic Biol Med 2012; 53: 20–29. 22609005 10.1016/j.freeradbiomed.2012.04.018

[16] Daugherty A , Dunn JL , Rateri DL , Heinecke JW . Myeloperoxidase, a catalyst for lipoprotein oxidation, is expressed in human atherosclerotic lesions. J Clin Invest 1994; 94: 437–444. 8040285 10.1172/JCI117342 PMC296328

[17] Nagra RM , Becher B , Tourtellotte WW , Antel JP , Gold D , Paladino T *et al* Immunohistochemical and genetic evidence of myeloperoxidase involvement in multiple sclerosis. J Neuroimmunol 1997; 78: 97–107. 9307233 10.1016/s0165-5728(97)00089-1

[18] Kettle AJ , Winterbourn CC . Myeloperoxidase: a key regulator of neutrophil oxidant production. Redox Rep 1997; 3: 3–15. 27414766 10.1080/13510002.1997.11747085

[19] Hawkins CL , Morgan PE , Davies MJ . Critical methods in free radical biology and medicine: quantification of protein modification by oxidants. Free Radic Biol Med 2009; 46: 965–988. 19439229 10.1016/j.freeradbiomed.2009.01.007

[20] Davis SS , Mikami DJ , Newlin M , Needleman BJ , Barrett MS , Fries R *et al* Heating and humidifying of carbon dioxide during pneumoperitoneum is not indicated: a prospective randomized trial. Surg Endosc 2006; 20: 153–158. 16333546 10.1007/s00464-005-0271-x

[21] Erikoglu M , Yol S , Avunduk MC , Erdemli E , Can A. Electron‐microscopic alterations of the peritoneum after both cold and heated carbon dioxide pneumoperitoneum. J Surg Res 2005; 125: 73–77. 15836853 10.1016/j.jss.2004.11.029

[22] Peng Y , Zheng M , Ye Q , Chen X , Yu B , Liu B . Heated and humidified CO_2_ prevents hypothermia, peritoneal injury, and intra‐abdominal adhesions during prolonged laparoscopic insufflations. J Surg Res 2009; 151: 40–47. 18639246 10.1016/j.jss.2008.03.039

[23] Sammour T , Mittal A , Delahunt B , Phillips AR , Hill AG . Warming and humidification have no effect on oxidative stress during pneumoperitoneum in rats. Minim Invasive Ther Allied Technol 2011; 20: 329–337. 21395459 10.3109/13645706.2011.556647

[24] Glew PA , Campher MJ , Pearson K , Schofield JC , Davey AK . The effect of warmed, humidified CO_2_ on the dissipation of residual gas following laparoscopy in piglets. J Am Assoc Gynecol Laparosc 2004; 11: 204–210. 15200776 10.1016/s1074-3804(05)60200-9

[25] Dindo D , Demartines N , Clavien PA . Classification of surgical complications – a new proposal with evaluation in a cohort of 6336 patients and results of a survey. Ann Surg 2004; 240: 205–213. 15273542 10.1097/01.sla.0000133083.54934.ae PMC1360123

[26] Buss IH , Senthilmohan R , Darlow BA , Mogridge N , Kettle AJ , Winterbourn CC . 3‐chlorotyrosine as a marker of protein damage by myeloperoxidase in tracheal aspirates from preterm infants: association with adverse respiratory outcome. Paediatr Res 2003; 53: 455–462. 10.1203/01.PDR.0000050655.25689.CE 12595594

[27] Mita H , Higashi N , Taniguchi M , Higashi A , Kawagishi Y , Akiyama K . Urinary 3‐bromotyrosine and 3‐chlorotyrosine concentrations in asthmatic patients: lack of increase in 3‐bromotyrosine concentration in urine and plasma proteins in aspirin induced asthma after intravenous aspirin challenge. Clin Exp Allergy 2004; 34: 931–938. 15196282 10.1111/j.1365-2222.2004.01968.x

[28] Van Der Vliet A , Nguyen MN , Shigenaga MK . Myeloperoxidase and protein oxidation in cystic fibrosis. Am J Physiol Lung Cell Mol Physiol 2000; 279: 537–546. 10.1152/ajplung.2000.279.3.L537 10956629

[29] Wu SM , Pizzo SV . α_2_‐Macroglobulin from rheumatoid arthritis synovial fluid: functional analysis defines a role for oxidation in inflammation. Arch Biochem Biophys 2001; 391: 119–126. 11414692 10.1006/abbi.2001.2408

[30] Cheong JY , Keshava A , Witting P , Young CJ . Effect of intraoperative warmed, humidified CO_2_ insufflation during abdominal surgery: a review. Ann Coloproctol 2017; 34: 125–137. 10.3393/ac.2017.09.26 PMC6046539 29991201

[31] Brokelman WJ , Holmdahl L , Bergström M , Falk P , Klinkenbijl JH , Reijnen MM . Heating of carbon dioxide during insufflation alters the peritoneal fibrinolytic response to laparoscopic surgery: a clinical trial. Surg Endosc 2008; 22: 1232–1236. 17943363 10.1007/s00464-007-9597-x

[32] Sammour T , Kahokehr A , Hayes J , Hulme‐Moir M , Hill AG . Warming and humidification of insufflation carbon dioxide in laparoscopic colonic surgery: a double‐blinded randomized controlled trial. Ann Surg 2010; 251: 1024–1033. 20485147 10.1097/SLA.0b013e3181d77a25

[33] Moehrlen U , Ziegler U , Boneberg E , Reichmann E , Gitzelmann CA , Meuli M *et al* Impact of carbon dioxide *versus* air pneumoperitoneum on peritoneal cell migration and cell fate. Surg Endosc 2006; 20: 1607–1613. 16823647 10.1007/s00464-005-0775-4

[34] Hazebroek EJ , Schreve MA , Visser P , De Bruin RW , Marquet RL , Bonjer HJ . Impact of temperature and humidity of carbon dioxide pneumoperitoneum on body temperature and peritoneal Morphology. J Laparoendosc Adv Surg Tech A 2002; 12: 355–364. 12470410 10.1089/109264202320884108

[35] Margulis V , Matsumoto ED , Tunc L , Taylor G , Duchenne D , Cadeddu JA . Effect of warmed, humidified insufflation gas and anti‐inflammatory agents on cytokine response to laparoscopic nephrectomy: porcine model. J Urol 2005; 174: 1452–1456. 16145469 10.1097/01.ju.0000173011.81396.12

[36] Sammour T , Mittal A , Loveday BP , Kahokehr A , Phillips AR , Windsor JA *et al* Systematic review of oxidative stress associated with pneumoperitoneum. Br J Surg 2009; 96: 836–850. 19591166 10.1002/bjs.6651

[37] Arsalani‐Zadeh R , Ullah S , Khan S , MacFie J . Oxidative stress in laparoscopic *versus* open abdominal surgery: a systematic review. J Surg Res 2011; 169: e59–e68. 10.1016/j.jss.2011.01.038 21492871

